# Trends in Liver Transplantation in Hepatitis C Virus–Infected Persons, United States

**DOI:** 10.3201/eid2203.151650

**Published:** 2016-03

**Authors:** Ryan B. Perumpail, Robert J. Wong, Andy Liu, Channa R. Jayasekera, Douglas T. Dieterich, Zobair M. Younossi, Aijaz Ahmed

**Affiliations:** Stanford University School of Medicine, Stanford, California, USA (R.B. Perumpail, A. Ahmed);; Highland Hospital, Oakland, California, USA (R.J. Wong);; Albert Einstein School of Medicine, Bronx, New York, USA (A. Liu);; California Pacific Medical Center, San Francisco, California, USA (C.R. Jayasekera);; Icahn School of Medicine at Mount Sinai, New York, New York, USA (D.T. Dieterich);; Inova Fairfax Hospital Center for Liver Diseases, Falls Church, Virginia, USA (Z.M. Younossi);; Inova Health System Betty and Guy Beatty Center for Integrated Research, Falls Church (Z.M. Younossi)

**Keywords:** liver, transplantation, hepatitis C virus, HCV, waitlist, infection, viruses, United States, trends

**To the Editor:** The Centers for Disease Control and Prevention and US Preventive Services Task Force recommend a one-time screening for hepatitis C virus (HCV) infection in adults born during 1945–1965 (birth cohort), a demographic group with a disproportionately high prevalence of HCV infection (*1*,*2*). However, some experts have warned against routine HCV screening of persons in the birth cohort, stating that this recommendation is based on unproven assumptions about the benefit of screening in reducing HCV-related mortality, given that only a minority of infected persons develop end-stage liver disease (ESLD) (*3*). To determine the relative effect of the birth cohort on HCV-related ESLD incidence in the United States, we analyzed trends in liver transplantation (LT) waitlist registrations and LT surgeries during 1995–2012. Using data from the United Network for Organ Sharing national registry, we evaluated birth cohort–specific (birth cohort vs. non–birth cohort) and etiology-specific (HCV vs. non-HCV) trends in LT waitlist registrations and LT surgeries performed in the United States during that 18-year period.

The proportion of HCV-infected persons born during 1945–1965 among all persons with LT waitlist registrations in the United States increased from 17.8% in 1995 to 35.2% in 2012 (Table). The highest proportion of LT waitlist registrations for HCV-related ESLD was for persons in the birth cohort and increased incrementally from 61.2% in 1995 to 90.5% in 2012. The proportion of LT waitlist registrations for HCV-related ESLD among persons younger than the birth cohort was 1.0% in 1995 and 3.6% in 2012; among persons older than the birth cohort, the proportion was 37.8% in 1995 and 5.9% in 2012.

**Table:** Liver transplant waitlist additions and liver transplant recipients, United States*

Transplant status	1995	2001	2006	2012
New waitlist additions				
Birth cohort† total	3,227 (47.4)	6,329 (62.3)	7,378 (71.9)	8,476 (77.0)
HCV	1,212 (17.8)	2,960 (29.1)	3,312 (32.3)	3,872 (35.2)
Non-HCV	2,015 (29.6)	3,369 (33.2)	4,066 (39.6)	4,604 (41.8)
Non–birth cohort total	3,583 (52.6)	3,830 (37.7)	2,878 (28.1)	2,350 (23.0)
HCV	767 (11.3)	818 (8.1)	505 (4.9)	408 (3.7)
Non-HCV	2,816 (41.3)	3,012 (29.6)	2,373 (23.2)	2,122 (19.3)
Total	6,810 (100.0)	10,159 (100.0)	10,256 (100.0)	11,006 (100.0)

Liver transplant recipients				
Birth cohort total	1,677 (48.9)	2,926 (63.7)	4,324 (71.2)	4,475 (78.1)
HCV	598 (17.4)	1,416 (30.8)	2,004 (33.0)	2,029 (35.4)
Non-HCV	1,079 (31.5)	1,510 (32.9)	2,320 (38.2)	2,446 (42.7)
Non–birth cohort total	1,751 (51.1)	1,667 (36.3)	1,747 (28.8)	1,256 (21.9)
HCV	396 (11.6)	393 (8.6)	309 (5.1)	208 (3.6)
Non-HCV	1,355 (39.5)	1,274 (27.7)	1,438 (23.7)	1,048 (18.3)
Total	3,428 (100.0)	4,593 (100.0)	6,071 (100.0)	5,731 (100.0)

*HCV, hepatitis C virus. †US adults born during 1945–1965.

Similarly, among LT recipients, the proportion of HCV-infected persons born during 1945–1965 doubled from 17.4% in 1995 to 35.4% in 2012 (Table). The proportion of LT surgeries performed for HCV-related ESLD among persons in the birth cohort increased from 60.2% in 1995 to 90.7% in 2012. Among persons younger than the birth cohort, the proportion of LT surgeries performed for HCV-related ESLD was 0.7% in 1995 and 5.0% in 2012; among persons older than the birth cohort, the proportion was 39.1% in 1995 and 4.3% in 2012.

During 1995–2012, the ratio of new LT waitlist registrations to LT surgeries performed for HCV-infected persons in the birth cohort remained unchanged at 1.9:2.0 despite the aging of this birth cohort. Overall trends in HCV-related LT waitlist registrations and LT surgeries stabilized during 2001–2012; the proportion of HCV-infected persons in the birth cohort increased, and the proportion of HCV-infected persons not in the birth cohort decreased.

To exclude the possibility that HCV-related ESLD has always simply affected persons 50–70 years of age, we performed a subanalysis examining the proportion of LT waitlist registrations and LT surgeries for persons 50–70 years of age in each year from 1995 through 2012. During this 18-year period, among persons 50–70 years of age, new HCV-related LT waitlist registrations increased from 43.9% to 93.0%, and LT surgeries performed increased from 47.1% to 86.2%. This finding suggests that persons born during 1945–1965 are a distinct birth cohort that is increasingly affected by HCV-related ESLD.

Although persons born during 1945–1965 make up an estimated 27% of the US population, they account for ≈75% of all HCV infections and 73% of HCV-associated deaths in the United (*1*). Our findings are consistent with those of an earlier modeling study by Davis et al. (*4*), which suggested that the age of persons with HCV-related cirrhosis and its complications will continue to increase.

Limitations of our study include inherent limitations of retrospective design and registry data. The designation of HCV infection and birth cohort status is based entirely on data entered into the database, which are not necessarily subject to cross-checking confirmatory measures. However, any errors in data entry that may have occurred are probably nondifferential. Despite these limitations, our analysis demonstrates that >90% of HCV-infected persons registered for LT or undergoing LT surgeries in 2012 were in the birth cohort.

Earlier diagnosis and preemptive cure of HCV infection with highly effective and safe direct-acting antiviral drugs may delay or reduce the need for LT among persons in the birth cohort (*5*). Testing and linkage to care for HCV-infected persons, particularly persons in the birth cohort, can be expected to reduce HCV-related illness and death (*1*,*2*). In response to the approval of higher efficacy antiviral drugs and rapidly rising liver failure–related death among this cohort (*6*,*7*), the use of HCV-infected donors has increased, resulting in truncated wait times for HCV-infected LT recipients in many regions (*8*), whereas HCV-uninfected persons are generally waiting considerably longer, often years, for HCV-uninfected donors (*9*). This phenomenon is another index of the extent of HCV-related ESLD in the United States.
